# History and Statistics of Ovariotomy, and the Circumstances under Which the Operation May Be Regarded as Safe and Expedient

**Published:** 1857-01

**Authors:** 


*History and Statistics of Ovariotomy, and the Circumstances under which the Operation may be regarded as Safe and Expedient; being a Dissertation to which the Prize of the Massachusetts Medical Society was awarded, May,* 1856. By GEORGE H. LYMAN, M.D. Boston: Printed by John Wilson & Son, 1856.

This is a well written and well printed monograph, of one hundred and forty-six pages. It contains a full historical account of the origin and practice of operations for the removal of the ovaria, with statistical tables presenting the results of three hundred operations. The numerical results of these operations are given as follows:—

'I.—Of the 300 cases, the operation was completed by the removal of the tumor in 208; which, excluding 4 not mentioned, gives us 70.27 in 100.

'The tumor could not be removed in 78; or 1 in $3^{31-39}$, or 26.35 in 100.

'The tumor was partially removed in 10; or 1 in $29^{3-5}$, or 3.37 in 100.

'The removal of the tumor is not mentioned in 4.

'II.—In 1 case the result is not stated; of the remaining 299 operations, 179 recovered, 120 died; or 1 in $2^{59-120}$, or 40.14 in 100.

'III.—Of the 208 cases in which the operation was completed, 119 recovered; or 57.21 in 100: 89 died, or 1 in $2^{30-89}$, or 42.78 in 100.

'IV.—The above gives us, therefore, 300 operations for the removal of ovarian disease, of which 119 only were successful *in the removal of the disease and the recovery of the patient;* or 1 in $2^{62-119}$, or 39.66 to 100—less than two-fifths.

'V.—Of the 78 cases in which the tumor could not be removed, 55 recovered from the operation; or 70.51 in 100: 22 died, or 1 in $3^{6-11}$, or 28.20 in 100; and in 1 the result is not given.

'VI.—Of the 10 cases in which the tumor was partially removed, 5 died, and 5 recovered from the operation.

'This does not include those cases in which the cyst or tumor was emptied or incised, or a tent introduced. Of the 88 cases included in these two sections, V. and VI. in which the operation remained unfinished, 27 died; or 1 in $3^{7-27}$, or 30.68 in 100.'

The following are the conclusions to which the author has arrived in relation to the propriety of resorting to the operation under given circumstances, viz.:—

'1. The mortality attendant upon Ovariotomy is no greater than it is after other capital operations.

'2. The mortality resulting from extensive incisions of the peritoneum is generally over estimated.

'3. Fully developed cystic disease of the ovarium tends rapidly to a fatal result.

'4. No method of treatment heretofore devised for it is so successful as extirpation; excepting, possibly, that by injection with iodine, of the results from which we have, as yet, insufficient statistics.

'5. The operation is unjustifiable in the early stages of the disease.

'6. After active development has commenced, with the supervention of constitutional symptoms, the sooner the operation is performed, the greater the chance of recovery.

'7. No rule can be laid down as to the length of the incision, other than the general one—that the shorter it is, the less the mortality; and that, therefore, the primary incision should always be small, and extended afterwards as may be necessary, according to the exigencies of each particular case.

'8. If, after the operation is commenced, extensive adhesions should be discovered, either the complete abandonment of the intended extirpation, or the attempt to cause suppuration and gradual contraction of the cyst, by means of a permanent external opening, are to be preferred to the division of the adhesions and completion of the operation as originally designed.'

